# Exploring the restorative benefits of work in smart working structures on vacations in small villages

**DOI:** 10.3389/fpsyg.2023.1232318

**Published:** 2023-12-19

**Authors:** Luigi Maffei, Antonio Ciervo, Raffaella Marzocchi, Massimiliano Masullo

**Affiliations:** SENS i-Lab, Department of Architecture and Industrial Design, University of Campania Luigi Vanvitelli, Aversa, Italy

**Keywords:** smart working, movable office, restorativeness, performance, prefabricated modular office, small villages, historic sites, water elements

## Abstract

**Introduction:**

Processes of redefinition of work, already in place in the pre-pandemic era, with the advent of COVID-19, have become widely required. A “model of work” that uses new technologies and the development of existing ones to improve workers’ performance and satisfaction has emerged. Smart working has changed how people work and, as a result, spaces must also change to support them. The use of prefabricated movable buildings (PMB) could represent an opportunity to create smart (and co-) working spaces in a regenerative contest. Small villages with historical, architectural, and naturalistic elements and slow-life features are potential platforms where vacation and work could merge easily. This paper aims to establish if working in movable offices, like the PMBs, which provide a high level of visual and acoustics interaction with the surroundings, and which is positioned in small villages’ squares, can affect workers’ sense of restoration and working performance.

**Methods:**

In a laboratory setting, in the SENS i-Lab, a videowall and a 3D spatial audio system were used to compare, in terms of restorativeness and self-reported cognitive performance, the effects of a high-rise building context of a City Business District (CBD), i.e. the control scenario, and of two typical squares of small Italian villages with historic buildings, without (HIS) and with water elements (HIS-W).

**Results:**

The findings of the experimental sessions showed that when working in close contact (visual and auditory) with historical or water elements, where life flows slowly, workers perceived a higher sense of restoration while job performance was unchanged.

**Discussion:**

Implementing smart working policies in small villages that encourage the use of energy-efficient prefabricated movable buildings and that offer a high level of visual and acoustic interaction with the surrounding environment may represent a promising strategy to foster the development of the local economy and contrast the depopulation of small villages, improving the worker well-being and the reducing the impact of their activities of the environment.

## 1 Introduction

Since the end of the last century, the phenomenon of the digital nomads (DNs), that is the possibility to work remotely from any location being free to travel the world while using portable computing technologies and broad Internet connectivity, was analyzed from a social life perspective. Makimoto and Manners (1997), studying digital nomads as early as 1997, envisioned a globalized world in which new technologies, new kinds of work arrangements, and a growing emphasis on entrepreneurial practices would radically change our lives, blurring distinctions between work, leisure, home, and travel. The COVID-19 pandemic has changed the perspective of work, expanding hugely the possibility of working remotely. Twenty years later, the digital nomad has evolved from a merely fictional character into a social figure of current work life.

Processes of redefinition of work, already in place in the pre-pandemic era, with the advent of COVID-19, have become widely required (Bonacini et al., 2021), and workers of organizations of different sectors (i.e., tertiary, education, professional services, and public administration) became involved in a new form of flexible work (Marino and Capone, 2021), namely, “smart working.” According to Rapisarda et al. (2021), smart working is based on four fundamental requirements: digital and remote online working (*Nature of the work* and *Technology*), a flexible achievement-based performance relationship structure no longer time-based performance (*Relationship structure*), and a suitable place to carry out one’s work (*Logistics*), and mainly aims to enhance the efficiency/effectiveness of the services provided and to increase workers’ satisfaction and autonomy (i.e., operating at the preferred time and in the preferred environment). Smart working can be considered an evolution of Telework (also referred to as telecommuting or remote work) which compared to smart working should be carried out on a regular basis and often tied to a certain work location (often their home or telework center; Rymkevich, 2018; Beckel and Fisher, 2022). For employees, smart working is a way to optimize schedules, save commuting time and costs, and achieve a higher quality of life. In 2021, Italy registered 33 million commuters, 22 million workers, and 11 million students (ISTAT, 2023). The workers’ commuters spend, on average, 70 euros per month and 72 min a day to reach their workplace, costs that could well be reduced by a permanent or quasi-permanent smart working schedule (Aksoy et al., 2023). Today increasingly, individuals and even entire start-up teams, especially in the creative sector, follow the idea of a location-independent style of working and living. Smart working has changed the way people work, and, as a result, spaces must also change to support them.

Performance on the job and workplace quality are closely related (Pasini et al., 2021), and productivity is positively impacted in environments where the worker may continuously regenerate physically and psychologically. Environments that support the process of renewing physical, psychological, and social capabilities that have been lost as a result of continual efforts to meet adaptive needs (known as “restoration”) are defined as restorative environments (Pasini et al., 2021). Beyond the physical perception of comfort (indoor air quality, noise, lighting, and thermal comfort; Bluyssen et al., 2011), other factors of the surrounding environment significantly affect the individuals. In particular, natural environments have been found to provide positive esthetic and emotional experiences (Ulrich, 1983), mitigate and prevent stress (Kaplan, 1995), and affect directed attention abilities (Berman et al., 2008). Among them, researchers have demonstrated the existence of strong correlations between pleasantness and wellbeing with green, i.e., trees and grass (Dzhambov and Dimitrova, 2014; Watts, 2017), water elements, i.e., fountains, waterfalls, streams, and water basins (Watts et al., 2009; Jeon et al., 2010; De Coensel et al., 2011), and their combination (Axelsson et al., 2014; Chau et al., 2018; Lugten et al., 2018; Chung et al., 2019). In built urban environments, the qualities and attributes of natural elements that people find appealing and esthetically pleasing can be used (Zhang et al., 2017; Scopelliti et al., 2019), or reproduced to promote psychological restoration (Berto et al., 2015; Masullo et al., 2021a). This is in line with the ART Theory (Kaplan, 1995) and other studies which state that performance benefits from exposure to environments that attract effortless involuntary attention and fascination. Due to the high potential of fascination, places with high historical and architectural value can also provide high restorative effects (Weber and Trojan, 2018). This is also confirmed by the recent studies of Wang et al. (2023) on typical cultural heritage sites in China and Masullo et al. (2021b) on Italian cloisters and Turkish Khan. For Galindo and Hidalgo (2005) and Hidalgo et al. (2006), cultural-historical and recreational places were considered more attractive than panoramic places and housing areas. Moreover, restorativeness was found to be a significant predictor of a place’s attractiveness. Comparing a historical site, an urban park, a panoramic promenade, and a shopping mall, Fornara and Troffa (2009) showed how historical sites can be perceived as fascinating, pleasant, and relaxing, as well as the urban park, while resulting in more restorative than panoramic promenade and a shopping mall. More in general, different types of urban spaces elicit several dimensions of mood (Rapuano et al., 2022), e.g., green parks induced greater feelings of calm than colorful parks and squares, while colorful parks were generally rated as more energizing than calming. In addition, different lifestyles affect individuals’ overall quality of life differently (Botta, 2016; Kim et al., 2019). Regarding the latter aspect, the slow living philosophy came to fruition through CittàSlow Movement, born in 1999 by the mayor of Greve in Chianti, and is now spread worldwide. It draws up a series of “good practices,” starting from the assumptions of Slow Food, that improve the quality of life in small villages. Slow life, therefore, refers to the health of citizens, quality of local food and respect for traditions and culture, and time spent enjoying the cultural and natural landscapes that the small villages offer (Parkins and Craig, 2006).

Small villages with historical, architectural, naturalistic elements and slow-life features are then a potential platform where vacation and work could merge easily. The increasing investments in their digital connectivity can then transform them into an excellent location for hosting smart workers and digital nomads. Nowadays, in these small villages and countryside (Bosworth et al., 2023), that suffer a strong phenomenon of depopulation, there are several ongoing projects that promote the realization of local smart working policies (Italian Ministry of Cultural Heritage, 2022). In this context, the way the smart/co-working spaces are realized assumes a strong importance (Bosworth et al., 2023).

Smart work reinforces the making of collaborative spaces in two ways: First, it disrupts the spatial divisions classified by codes of land uses and space uses (Hu, 2020). It introduces a smart design paradigm that incorporates this spatial disruption and creates collaborative spaces to enable innovative knowledge production. Second, the collaborative space further goes beyond a physical dimension, integrating the ‘space of flow’ and the ‘space of place’ (Castells, 2010) to capture the spatiality in a network society, in knowledge production, consumption, and sharing. At the same time, co-working space definition refers to “flexible, shared, rentable, and community-oriented workspaces occupied by professionals from diverse sectors” that are “designed to encourage collaboration, creativity, idea sharing, networking, socializing, and generating new business opportunities for small firms, start-ups, and freelancers” (Fuzi, 2015). Orel (2019) studied the co-working spaces and concluded that co-working helps address the challenges associated with work–leisure balance and is seen as a venue for socializing and even wellbeing. In addition, the concept of the co-working space helps to self-discipline and retain work-related routines (Reichenberger, 2018; Cook, 2020).

Smart/co-working spaces should be, then, flexible, shared, rentable, and community-oriented. They should be designed to encourage tourism, collaboration, creativity, idea sharing, networking, socializing, and generating new business opportunities for small firms, start-ups, and freelancers (Bosworth et al., 2023). The restorative impact of historical and architectural elements (Galindo and Hidalgo, 2005; Hidalgo et al., 2006) on smart workers and digital nomads could be emphasized with the realization of temporary rooms, or offices, for smart working located both inside the main tourist attractions (museums, convent complexes, historic buildings) and in symbolic outdoor locations (central squares, belvederes; Chevtaeva and Denizci-Guillet, 2021).

Furthermore, it should be noted that despite facing depopulation, a lack of employment opportunities, and a lack of essential services, many small Italian villages representing 70% of Italian cities and 11 millions of residents have relevant regenerative potential and may be appropriate for smart/co-working applications (Greenreport, 2022; ISTAT, 2022). In addition, the retrofit actions for energy and to optimize occupants’ wellbeing for Italian historic buildings (for example, for smart/co-working application) located in small historical villages cause frequently disadvantages related to structure and existing regulations constraints (Galatioto et al., 2019). In particular, the constraints of the existing regulations for the preservation of the historic value of buildings make energy refurbishment very challenging because the application of passive and active energy strategies is often in contrast with architectural features in most cases, and the best available technologies are not applicable since they are in contrast with the conservation of their historic value and occur frequently difficulty in installing renewable energy sources due to architectural law constraints that are applied often not only on the building but also on the district where they are located (Galatioto et al., 2019). Therefore, creating a self-sustaining, eco-friendly, modular, and flexible mobile building for smart/co-working could aid small communities’ social and economic revival with high regeneration potential.

As part of a wide research project aimed to design and prototype a prefabricated movable building (PMB), different design solutions were studied for smart/co-working and optimized for occupants’ wellbeing, energy performance, and integration with outdoor architectural/historical/landscape elements. Modular solutions of PMBs, self-sustaining in energy use, renewable energy-based, eco-friendly, and flexible in the setup were also proposed (Castanò et al., 2022; Maffei et al., 2022, 2023). The proposed PMB does not require interventions on the existing heritage for installation and therefore represents a sustainable solution in all historical places subject to regulatory constraints. The proposed PMB mobile office is suitable for temporary uses and, therefore, removable when the use of the area it occupies is required. Regarding the construction and transformability of the unit, a “kit-of-parts” system was developed, and they can be juxtaposed in several different configurations according to the location of their installation. The floor area of different PMBs’ configurations ranges from about 15 m^2^ to 100 m^2^, and their size can be modulated proportionally (e.g., the floor area of the PMB compared to the area of the square is about 5%) to those of the small village squares which can range, e.g., in the Campania Region (Italy), from about 300 m^2^ to 3,000 m^2^. The PMB offices are equipped with large smart windows and natural ventilation grilles. The smart window’s vast size and the natural ventilation grilles serve to provide a high level of visual and acoustics interaction with the landscape and soundscape to offer potential regenerative benefits to the worker through a multisensory experience related to the context in which the office is located (Browning and Ryan, 2020).

Although the prefabricated movable building design has been tested in terms of energy efficiency, economic benefit, and environmental impact under different configurations (Maffei et al., 2023), it is still not investigated if these proposed temporary office solutions can improve the occupant wellbeing, enhance, or keep constant the general working performances. To this aim, positioning virtually a PMB office in different surrounding contexts and simulating the physical stimuli at the worker position, this study aims to establish if small villages’ squares contexts can affect workers’ sense of restoration and working performance, through the verification of the following hypotheses:

*H1*: Working in movable offices, such as the PMBs, which are positioned in small villages’ squares makes the working experience more restorative than in traditional offices.

*H2*: Working in movable offices, such as the PMBs, which are positioned in small villages’ squares influences the level of attention, concentration, and distraction of the workers.

## 2 Materials

The first part of this section describes the characteristics of the three sites selected to contextualize the office during the experimental sessions, and the procedures and tools used to record and reproduce the audio and visual stimuli in the laboratory. The second part presents the questionnaires administered to participants.

### 2.1 Audio and visual stimuli from the context

**Figure 1:**
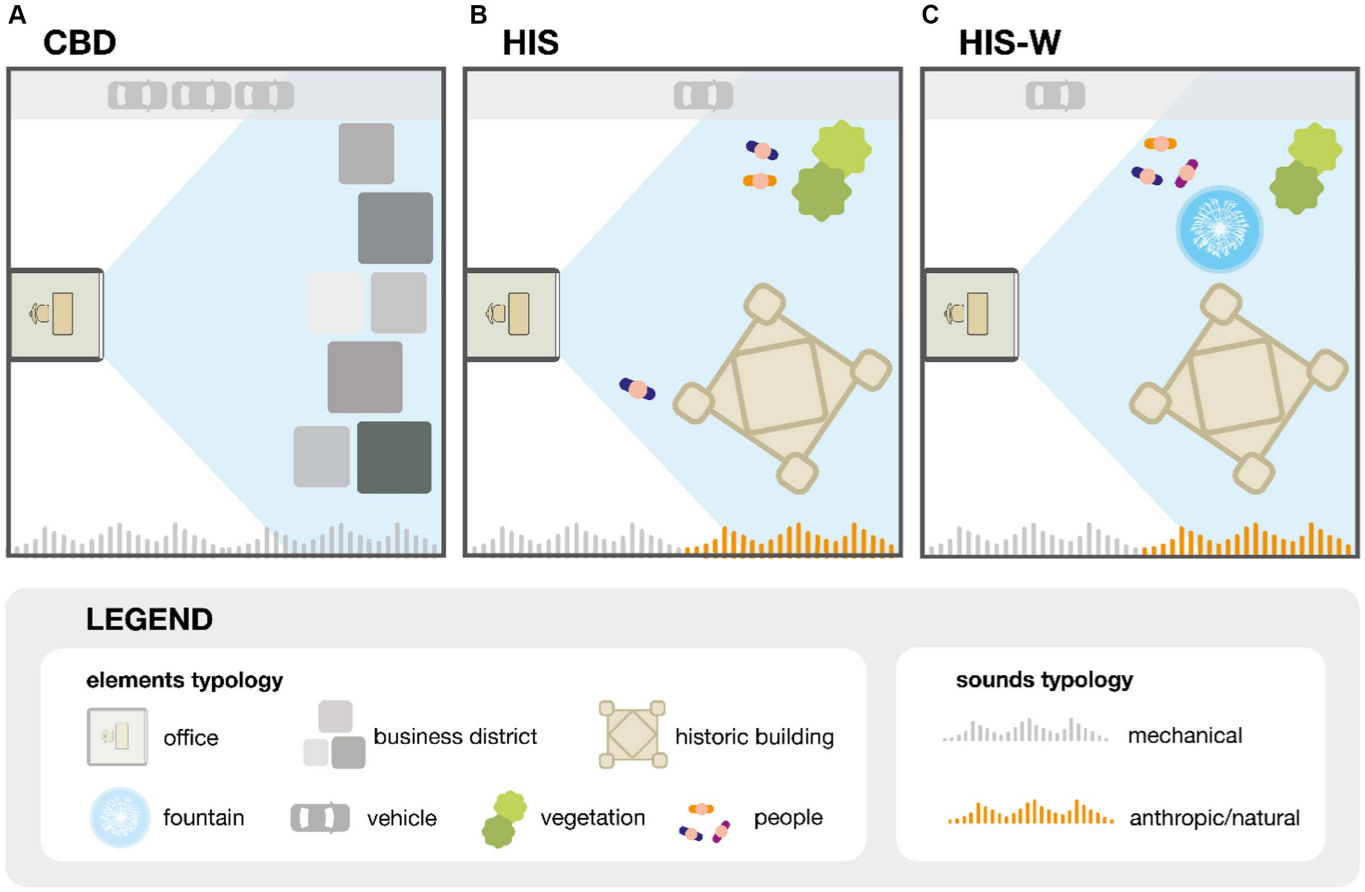
Schematic representation of the three scenarios analyzed: **(A)** CBD, **(B)** HIS, and **(C)** HIS-W.

Inside the test room of the SENS i-Lab, three different experiential reality scenarios were simulated by manipulating the view from the office windows and the sound at the worker position. The contexts consist of a control scenario, represented by a high-rise building of a large city’s business district (CBD) and two typical squares of small Italian villages with historic buildings, without (HIS) and with water elements (HIS-W). In particular, the CBD is used as a benchmark to compare the workers’ restorative effect and level of attention, concentration, and distraction with respect to the cases HIS and HIS-W. The authors collected each scenario’s visual and audio material through on-site recordings. In particular, the external view’s video captures were carried out using an iPhone 13 Pro with 4 K (3,840 × 2,160 pixels) resolution, positioned on a stand. At the same time, the audio recordings were carried out by a SQobold combined with Binaural Headset II. Figure 1 reports the schematic representation of the three scenarios analyzed: (a) CBD, (b) HIS, and (c) HIS-W. In particular, for each scenario analyzed, Figure 1 shows the elements that characterize each simulated scene, the window view of the worker (highlighted in light blue), and the sound typology reproduced during the experiment.

**Figure 2:**
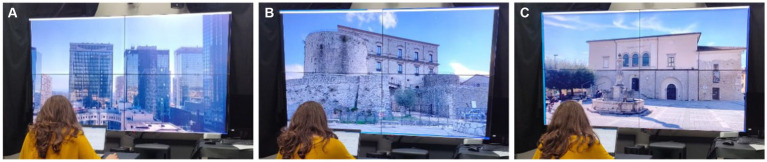
Images of the tests carried out in the SENS i-Lab test room: **(A)** CBD, **(B)** HIS, and **(C)** HIS-W scenarios.

Figure 2 reports the images of the tests carried out in the SENS i-Lab test room for the three different scenarios. A short description of the characteristics of the audio-video material used as stimuli in the laboratory is presented below:

1. CBD: The business district of the city of Naples (Italy) hosts offices of institutions and universities, and it is the headquarters of the city’s most important commercial and financial companies. Thirty min of audio and video recording were carried out at tenth floor office of a high-rise building in the district. The view of other high-rise buildings dominates the outdoor visual context of the office, while the video shows an almost static scene (Figure 2A). The auditory context is characterized by the noise coming from the outdoors and the adjacent rooms and corridors. All the recordings were performed with closed windows, no workers in the room, and all other devices (e.g., computers, smartphones, and printers) and the air-conditioning system turned off. The A-weighted sound equivalent level of the recorded was about 38 dB(A).
2. HIS: The first scene reproducing a small Italian village square (HIS) is Portello Square, a small square of the Teggiano Municipality (Province of Salerno, Italy) faced the ancient castle of the village (Figure 2B). This Castle, Macchiaroli Castle, was selected as the view out the window of the PMB office for the HIS scenario. Thirty min of video recording shows a very peaceful place with a few older people walking slowly through the square and rare car pass-by. The auditory scene is uneventful and characterized by natural sounds, bells ringing, people chatting, and a generally low and far background noise due to cars and motorcycles.
3. HIS-W: The second scene reproduces a small Italian village square in which there is the presence of a water element (HIS-W). The square is San Martino Square in the Cerreto Sannita municipality (Province of Benevento, Italy; Figure 2C). The view out from the window of the office consists of a historical palace (Palazzo del Genio) and a fountain (Fontana dei Delfini). Thirty min of video recording shows a scenario with some human activities with older people sitting outside a Café and others crossing the square. Traffic noise is still not excessive but higher than HIS.

To simulate the effects of sound transmission loss of PMB, the characteristics of the office façade consisting of a wide smart window and ventilation grilles were used to generate a one-third octave band filter and applied to the original recordings. The two resulting filtered A-weighted sound equivalent levels were about 31 dB(A) for HIS and 38 dB(A) per HIS-W. Finally, to control and simulate realistic working conditions, in the SENS i-Lab test room, the air-conditioning system was used to keep the same microclimatic condition during the experimental session. On the other hand, a laptop was given to the participants to carry out their activities. The background noise due to the air-conditioning system and the laptop was about 38 dB(A). This means that the same background noise characterized all experimental conditions.

### 2.2 Questionnaire

Three different questionnaires were used. A general information questionnaire investigated the participant’s age, gender, educational qualification, occupation, daily working hours, preferred type of vacation destination, and whether they would like to go more on vacation. Moreover, two different scales (or partial scales) investigated two main aspects of the working experience: self-reported cognitive performances and perceived sense of restoration.

#### 2.2.1 Self-reported cognitive performance

Workers’ self-reported cognitive performance was measured using a three-item questionnaire selected from the five-item concentration subscale of the Checklist Individual Strength (Vercoulen et al., 1994). Workers were asked to what extent they agreed with three statements after their working experience in the test room. Statements included “*I could concentrate well during working*” (Concentration), “*I had troubles keeping my attention on working*” (Attention), and “*During working my thoughts easily wandered*” (Distraction). The statements were rated on a five-point Likert scale ranging from one (strongly disagree) to five (strongly agree). The third negative item was re-coded, and the average score was obtained so that high scores reflect great attention.

#### 2.2.2 Perceived restorativeness scale

The Italian version of the Perceived Restorativeness Scale proposed by Pasini et al. (2014), PRS-11, was used to evaluate the workers’ restoration related to the use of PMB offices. The PRS-11 is based on four main components of restorativeness: (i) *Fascination*, which refers to how an environment might attract the involuntary attention of a person; (ii) *Being-Away*, which refers to how an environment causes a person to feel free from everyday demands and obligations; (iii) *Coherence*, which refers to how an environment is perceived as organized or not; (iv) *Scope* that refers to how an environment offers the possibility of exploration, including the immediate surroundings and the areas that are out of sight or imagined.

## 3 Methodology

The outline of the experimental design is discussed in section 3.1, the details of the participants are reported in section 3.2, and the procedure performed is described in section 3.3.

### 3.1 Experimental design

The experimental session was conducted in the SENS I-Lab of the Department of Architecture and Industrial Design of the University of Campania “Luigi Vanvitelli” where the CBD, HIS, and HIS-W scenarios were reproduced using a videowall. The system, consisting of four LCD panels KVD5521B 55” Full HD resolution (1920 × 1,080 pixels), was used to recreate the office window by playing and viewing the video registered on-site. In the test room, Astro Spatial Audio (which combines Spatial Sound Wave (SSW) technology with SARA II Premium Rendering Engine) was used to simulate the sound of each simulated scenario. The HVAC system was used to provide control of the microclimatic experimental conditions at a temperature of 20°C with a dead band of ±1°C and relative humidity of 50% with a dead band of ±10%. The HVAC return and supplied air fan were set at a speed of 30%. The lighting control condition was also guaranteed through the LED lighting system installed in the test room, consisting of six ceiling-mounted LED lighting dimmable luminaires, able to range the illuminance level between 50 and 800 lux on a reference plane at 0.75 m to the floor and the correlated color temperature (CCT) of the emitted light between 3,000 and 5,800 K. For the entire duration of the test (30 min), the illuminance level on the desk was 350 lux with a color temperature of 4,000 K.

### 3.2 Participants

*A priori* analysis of statistical power and effect size was carried out to obtain enough statistical validity of the results. The power analysis was computed using G-Power software (Faul et al., 2007; Faul et al., 2009) for ANOVA repeated measure test. The pre-defined effect size of (f) of 0.4, power of the test (1 − β) of 0.95, and significant level (α) of 0.05 were used to calculate the minimum sample size for the experiment. To increase the effect size, the authors decided to increase the number of participants to 30 subjects. Thirty people attended the experiment voluntarily. They were equally distributed in age: 10 from 18 to 25 years; 10 from 26 to 35 years; and 10 from 36 to 65 years. Forty percent of the participants were females. Participants were Master/PhD students, administrative workers, and researchers/professors of the Department of Architecture and Industrial Design of the University of Campania ‘Luigi Vanvitelli’. Considering the educational level, nine participants had a high school degree (30.0%), four had a bachelor’s degree (13.3%), eleven had a master’s degree (36.7%), and six had a PhD (20.0%). Participants self-reported they worked less than 5 h a day (16.6%), from 5 to 8 h a day (36.7%), from 8 to 10 h a day (26.7%), and from 10 to 12 h a day (20.0%). Concerning their preference to spend their holidays, all the participants self-reported would like to go more on vacation. In total, 40.0% of them prefer as destination a big city, 26.6% the sea, 16.7% the mountain, and 16.7% small villages. All 30 participants self-reported would like to go more on vacation. All subjects were in good health and gave informed consent about their participation in the study after being told about the experiment’s purpose and process.

### 3.3 Procedure

The participants were asked to answer the general information questionnaire and, after more than 24 h, were invited to “SENS i-Lab” to attend the experimental session. The session consisted of three sub-sessions where the three experiential reality scenarios (CBD, HIS, and HIS-W) were reproduced in the test room. Once the participants read and signed the informed consent, the experimenter instructed them about their duties. They were invited to have a look at the PMB project, which represents their imaginary working place. Then, they were invited to sit at a desk at the center of the test room, with the sight toward the video wall, and were asked to perform their daily activities (laptop work, reading, and writing). Each sub-session lasted 30 min and was administered in a randomized sequence. During the last 3 min of each session, the participants were asked to answer the question on self-reported cognitive performance and perceived restorativeness. Sub-sessions were interspersed by 30 min breaks.

## 4 Results

After verifying the respect of the ANOVA assumptions and identifying potential outliers, and missing values (Tabachnick and Fidell, 1996), several repeated measure (RM) one-way ANOVAs were prepared. In particular, to compare the cognitive performance in the different experimental conditions, four different one-way RM-ANOVAs that treated the scenario as a three-level within-subject factor (CBD, HIS, and HIS-W) were carried out on the averaged values of the self-reported cognitive performance (partial-SCP) questionnaire and on each of the items of the scale: attention (ATT), concentration (CONC) and not-distraction (NOT-DIS). The results showed that neither the partial-SCP nor their items were found to be different from each other. In particular, this result shows that working (laptop work, reading, and writing) inside offices that offer high levels of visual and acoustic interactions with surroundings, such as the PMBs, in movable structures located in small villages’ squares where the life rhythms are slow, and in front of historical buildings and close to water elements, does not alter the working performances respect to work in large city’s traditional office (CBD).

These results can be explained by the fact that external factors affecting cognitive performance are generally loud/intense and/or are poor in quality. Jahncke et al. investigated noise levels varying from about 30 to 60 dB(A) due to typical background office noise (e.g., air-conditioning and PC) with the additional telephone and voice signals (Jahncke et al., 2011), while Monteiro et al. investigated noise levels ranging from about 45 to 68 dB(A) composed of environmental noise of fast food with alarm sounds (Monteiro et al., 2018). Both found that decreases were found in performing both mental and manual activities as the sound levels increased. On the other hand, at noise levels as low as 40 dB, the negative connotation of external road traffic noise can be responsible for the decline in working performance (Müller et al., 2023). This means that less frequent negative events (e.g., cars or motos pass-by and sirens) combined with natural or anthropic sounds may induce a positive emotional state in the individuals without draining attentive sources. In terms of the effect of visual stimuli, while those related to the brightness of the surroundings (Hvass et al., 2021; Masullo et al., 2022) and possible glare effects due to the sun (Rodriguez et al., 2016) have been mis-considered in this experiment, those related to the quality of the vision were found not to influence the working performances (e.g., distract them significantly from work). Considering the combination of the visual and auditory stimuli, De Korte et al. evaluated that the subjects who work in an open office environment were exposed to different types of visual or auditory distractions (walking past the visual field of the subject and telephone conversations within hearing distance). Their results showed that participants’ performance, concentration, and emotion were not affected while performing a simple computer task (De Korte et al., 2007).

On the other hand, to compare the perceived restorativeness during the working activities, several one-way RM-ANOVAs that treated the scenario as a three-level within-subject factor (CBD, HIS, and HIS-W) were carried out on the PRS-11 scale and each single item: *Fascination*, *Being-Away*, *Coherence,* or *Scope*. The four one-way RM-ANOVAs were conducted to determine whether perceived restorativeness levels differed across the working conditions.

**Figure 3:**
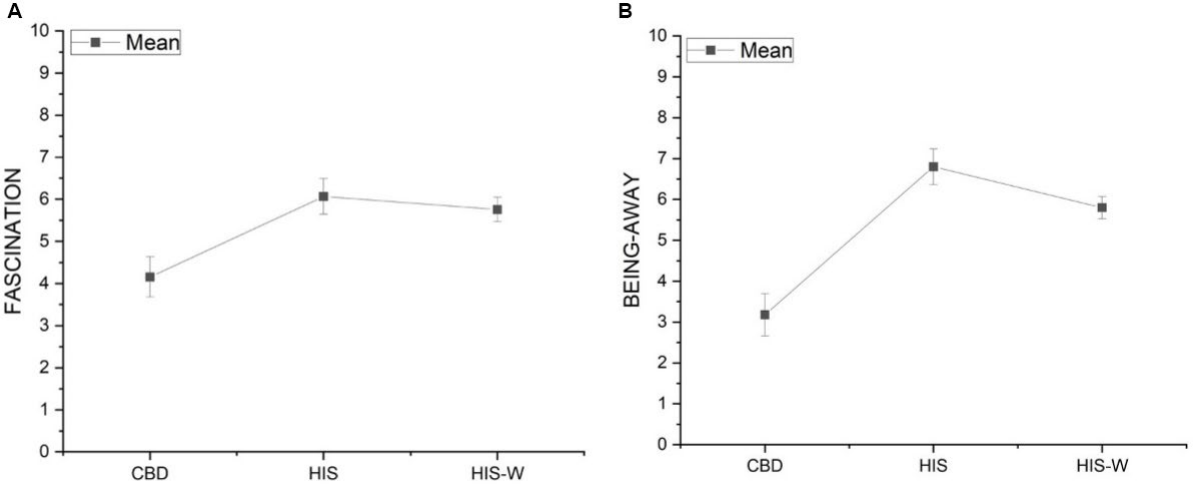
Marginal means and standard error of the *Fascination* **(A)** and *Being-Away* **(B)** components in the three experimental scenarios.

The results show that a statistically significant difference exists [*F*(2,58) = 8.87, *p* < 0.01] for the component *Fascination*. *Post-hoc* Tukey’s tests revealed that this component was significantly higher in participants while working in the Scenarios HIS (mean = 6.07) and HIS-W (mean = 5.76) than CBD (mean = 4.16; Figure 3A). Similarly, a statistically significant difference exists [*F*(2,58) = 24.89, *p* < 0.0001] for the component *Being-Away*. Post-hoc Tukey’s tests, again, revealed that the *Being-Away* was significantly higher in participants while working in the Scenarios HIS (mean = 6.80) and HIS-W (mean = 5.80) than CBD (mean = 3.18; Figure 3B).

**Figure 4:**
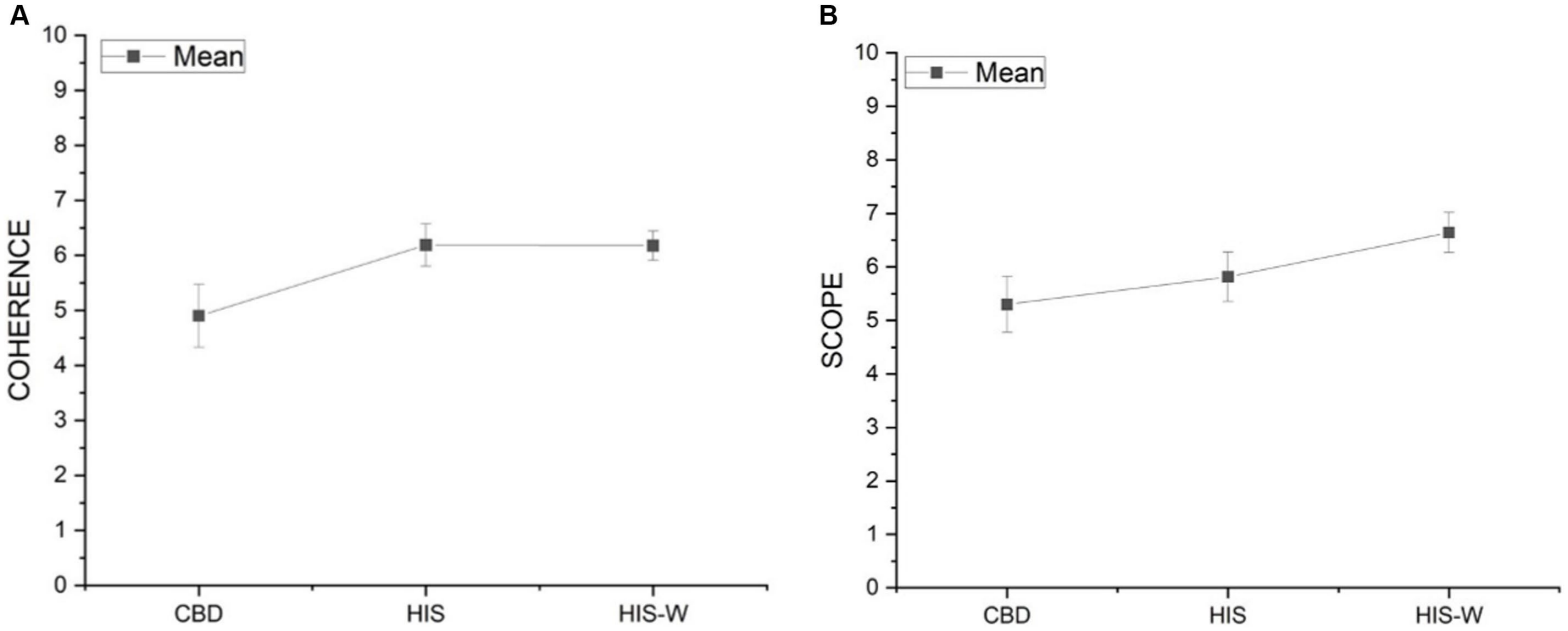
Marginal means and standard error of the *Coherence* **(A)** and *Scope* **(B)** components in the three experimental scenarios.

A less but still statistically significant difference resulted from the *Coherence* [*F*(2,58) = 3.72, *p* < 0.05] and *Scope* [*F*(2,58) = 3.26, *p* < 0.05]. *Post-hoc* Dunnet’s tests revealed that the *Coherence* was significantly lower in participants while working in the Scenarios CBD (mean = 4.90) than HIS (mean = 6.19) and HIS-WS (mean = 6.18; Figure 4A). Again, post-hoc Dunnet’s tests revealed that *Scope* was significantly lower in participants while working in the Scenarios CBD (mean = 5.30) than HIS-W (mean = 6.64; Figure 4B).

**Figure 5:**
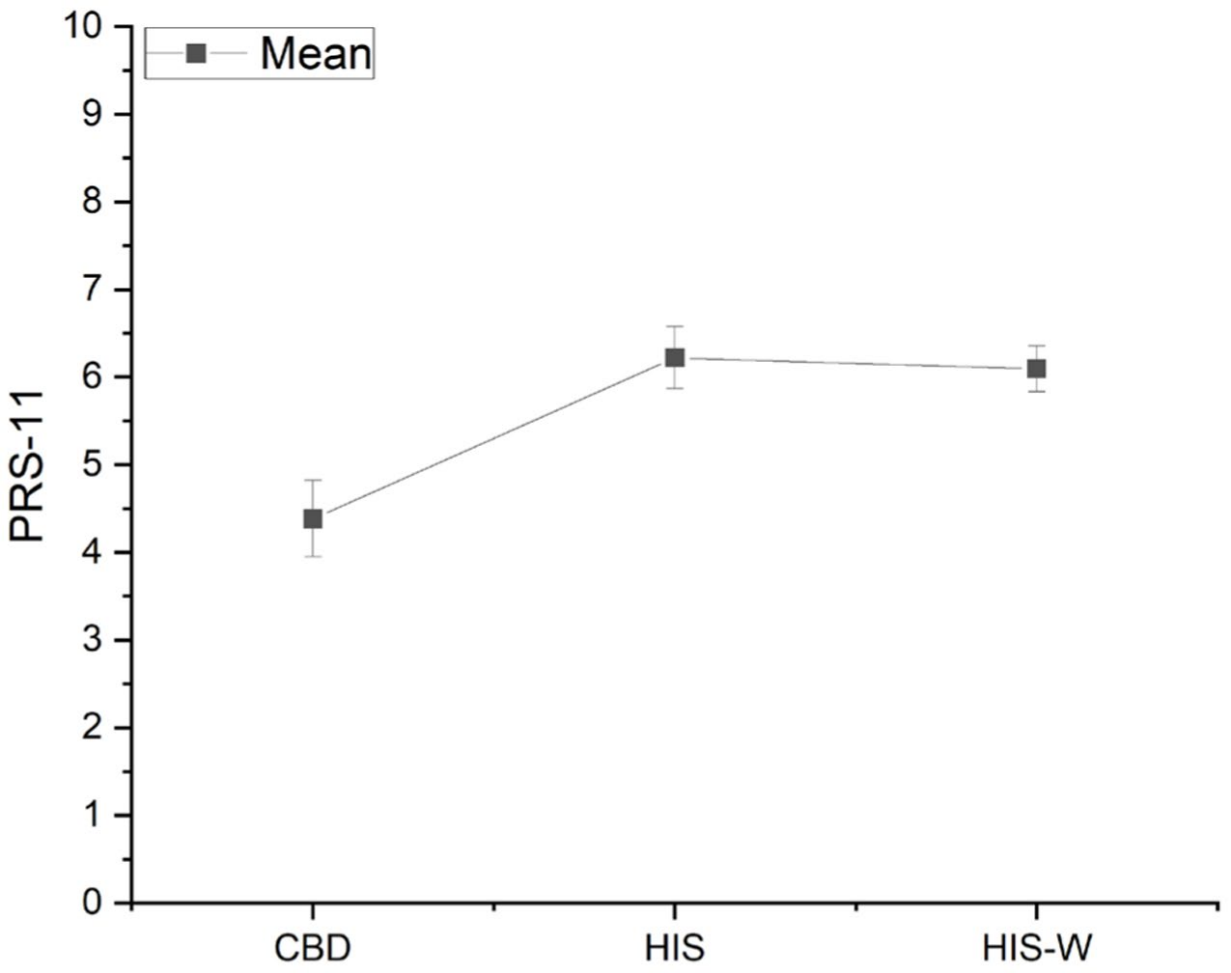
Marginal means and standard error of the *Perceived Restorative Scale (PRS-11)* in the three experimental scenarios.

The results on the complete PRS-11 scale still show a statistically significant difference existing [*F*(2,58) = 12.59, *p* < 0.0001]. *Post-hoc* Tukey’s tests revealed that perceived restoration was significantly higher working in the HIS (mean = 6.21) and HIS-W (mean = 6.09) than CBD (mean = 4.38; Figure 5).

The results can be explained by the fact that the restorative benefits obtained by workers are significantly higher when subjected to positively evaluated visual (historical buildings, vegetation, and fountain) and auditory stimuli (natural or anthropic sounds). The results about the HIS and HIS-W scenarios are in line with the previous literature which associates to this kind of context an increasing of two of the sub-dimensions of the PRS-11, the Fascination and Being-Away. A possible explanation is that, respectively, there is a “natural” attractiveness of vision toward the view of the historical buildings, as well as to listen to natural or anthropic sounds, and the contexts of the small villages foster a sense of being away from the routine working environment.

## 5 Conclusion

Temporary offices have been proposed as a possible solution to fight depopulation and the low economic level of small villages and countryside, to limit the concentration of environmental pollutants in urban areas, and to foster slow and more sustainable lifestyles. To this aim, the prefabricated movable building proposed by Maffei et al. (2023) was designed and tested under different configurations to guarantee energy-efficient solutions, enhance the local economy, and reduce the environmental impact. However, the possibility of access to new work modalities cannot neglect the importance of understanding the influence the surrounding environment can have on the workers.

This means that working in close contact (visually and auditory) with historical or water elements (e.g., historical buildings and fountains), where the life stream slowly, the workers’ wellbeing, improved, as shown by the increase of the restorativeness perception. This is mainly due to the rise in *Fascination* and *Being-away* of the visual contexts. At the same time, the research proves that the slow and quiet activities occurring in the squares of the small villages, just in front of the working position, do not influence working performance, concentration, and attention.

## Data availability statement

The raw data supporting the conclusions of this article will be made available by the authors, without undue reservation.

## Ethics statement

Ethical review and approval were not required for this study, in accordance with the local legislation and institutional requirements. However, the study was conducted in accordance with the Declaration of Helsinki and all the participants provided a written informed consent to participate in this study.

## Author contributions

LM, AC, and MM: conception and design of the study. AC: measurements. AC and RM: material collection, experiment setup, experimental sessions, writing the first draft of the manuscript, and data collections. RM: graphics and visualization. MM: statistical analysis. LM and MM: writing and reviewing. All authors contributed to the article and approved the submitted version.
